# Retraction: MiR-184 retarded the proliferation, invasiveness and migration of glioblastoma cells by repressing stanniocalcin-2

**DOI:** 10.3389/pore.2024.1611767

**Published:** 2024-03-28

**Authors:** Pathology & Oncology Research Editorial Office

Following publication, concerns were raised on the PubPeer platform regarding the validity of the data in the article. An investigation by the Editorial Office, conducted in accordance with Pathology and Oncology Research policies, was unable to confirm the validity of the data presented and as such cannot confirm its reliability. The article is therefore retracted.

This retraction was approved by the Editor-in-Chief of Pathology and Oncology Research. The authors received communication regarding the retraction but did not respond. The communication has been recorded by the publisher.

Pathology and Oncology Research would like to thank the concerned reader who contacted us regarding the published article.

